# Association between oxidative DNA damage and the expression of 8-oxoguanine DNA glycosylase 1 in lung epithelial cells of neonatal rats exposed to hyperoxia

**DOI:** 10.3892/mmr.2015.3339

**Published:** 2015-02-12

**Authors:** LINLIN JIN, HAIPING YANG, JIANHUA FU, XINDONG XUE, LI YAO, LIN QIAO

**Affiliations:** Department of Pediatrics, Shengjing Hospital of China Medical University, Shenyang, Liaoning 110004, P.R. China

**Keywords:** bronchopulmonary dysplasia, DNA damage, hyperoxia, neonatal, 8-oxoguanine DNA glycosylase 1

## Abstract

Previous studies have demonstrated that oxidative stress-induced lung injury is involved in the occurrence and developmental process of bronchopulmonary dysplasia (BPD). The present study assessed whether oxidative DNA damage occurs in the early stages of hyperoxia-induced BPD in neonatal rats and evaluated the expression and localization of the DNA repair gene, 8-oxoguanine DNA glycosylase 1 (OGG1), upon exposure to hyperoxia. Neonatal rats and primary cultured neonatal rat alveolar epithelial type II (AECII) cells were exposed to hyperoxia (90% O_2_) or normoxia (21% O_2_) and the expression levels of 8-hydroxy-2′-deoxyguanosine (8-OHdG) in the lung tissues and AECII cells were determined using a competitive enzyme-linked immunosorbent assay. DNA strand breaks in the AECII cells were detected using a comet assay. The expression and localization of the OGG1 protein in the lung tissues and AECII cells were determined by immunofluorescence confocal microscopy and western blotting. The mRNA expression levels of OGG1 in the lung tissues and AECII cells were determined by reverse transcription polymerase chain reaction. The expression of 8-OHdG was elevated in the hyperoxia-exposed neonatal rat lung tissue and the AECII cells compared with the normoxic controls. The occurrence of DNA strand breaks in the AECII cells increased with increasing duration of hyperoxia exposure. The protein expression of OGG1 was significantly increased in the hyperoxia-exposed lung tissues and AECII cells, with OGG1 preferentially localized to the cytoplasm. No concomitant increase in the mRNA expression of OGG1 was detected. These results revealed that oxidative DNA damage occurred in lung epithelial cells during early-stage BPD, as confirmed by *in vitro* and *in vivo* hyperoxia exposure experiments, and the increased expression of OGG1 was associated with this process.

## Introduction

Bronchopulmonary dysplasia (BPD) is a common and serious complication in premature infants born at a gestational age of <29 weeks (1). Among low birth weight infants (<1,500 g), the incidence of BPD approaches 43% (2), and ~50% of children with BPD are rehospitalized due to respiratory distress during early childhood, particularly in cases of concomitant respiratory syncytial viral infection (3). Long-term dysplastic diseases of the respiratory and nervous system are associated with a diagnosis of BPD in infancy and can persist into adolescence and adulthood, compromising the individual’s quality of life and resulting in substantial medical costs (4).

Clinical studies have identified several risk factors associated with the occurrence of BPD in premature neonates, including hyperoxia, ventilator-induced pulmonary injury and antenatal infection (5,6). These various factors are considered to act in a cumulative and synergic manner, causing early inflammation and lung injury, and leading to fibrosis and abnormal maturation processes (7). The mechanism underyling BPD remains to be fully elucidated, however, oxidative stress is important in the occurrence and development of BPD. Reactive oxygen species (ROS) induce lung injury and are a primary contributor to the pathogenesis of BPD (8,9). Our previous study demonstrated that oxidative stress-induced lung injury occurred during the development of BPD in a rat model of neonatal hyperoxia (10,11), consistent with other previous studies (12,13).

ROS induces significant, variable DNA damage, which can result in a loss of normal cellular functioning (14–16), unless cells activate timely DNA repair pathways (17). Oxidative DNA damage is repaired predominantly by the base excision repair (BER) pathways (15,16). In mammalian cells, 8-oxoguanine DNA glycosylase 1 (OGG1) is important in BER pathways (18,19). Overexpression of OGG1 in pulmonary artery endothelial cells reduces xanthine oxidase-induced mitochondrial DNA damage and cell apoptosis (20). By contrast, OGG1 knockdown by small interfering RNA in pulmonary artery endothelial cells, delays xanthine oxidase-induced DNA damage repair by mitochondria and increases the rate of cell apoptosis (21).

A previous study has suggested that OGG1 functions to antagonize oxidative DNA damage (22). However, few studies have examined whether lung epithelial DNA damage occurs during the process of hyperoxia-induced BPD in neonatals (23) or whether this damage is associated with OGG1. The present study examined the association between DNA damage in lung epithelial cells and OGG1 in a neonatal rat model of hyperoxia-induced BPD.

## Materials and methods

### Animals and hyperoxia exposure

A total of 20 pregnant Wistar rats (200–220 g) were purchased from the Center for Experimental Animals of China Medical University (Shengyang, China). All animal procedures were reviewed and approved by the Laboratory Animal Ethics Committee of China Medical University. The pups (n=80) were delivered naturally at full-term gestation (22 days). All of the rats were maintained in pathogen-free conditions and housed in a temperature- and humidity-controlled environment. They were all subjected to a 12 h light/12 h dark cycle and were given *ad libitum* access to food and water. The newborn rats from 12 litters were randomly assigned to either a hyperoxia-exposed group (90% O_2_) or a normoxia (21% O_2_) control group, from the day of birth. Inhaled oxygen concentrations were measured continuously using an oxygen analyzer equipped with a strip-chart recorder (model 572; Servomex, Co., Norwood, MA, USA). Humidity levels were maintained between 60 and 70%. Nursing rat dams were exchanged every 24 h between the hyperoxic and normoxic chambers to avoid oxygen toxicity and to provide equivalent nutrition to all pups. The chambers were opened for 10 min each day to weigh the pubs and for cage cleaning. Following 1, 2, 3, 5 or 7 days of exposure, the pups were sacrificed by abdominal aorta disconnection under intraperitoneal (i.p.) anesthesia (0.6 ml/100 mg 5% chloral hydrate; Sigma-Aldrich, St. Louis, MO, USA) and the lungs were harvested for subsequent analyses.

### Neonatal rat alveolar epithelial type II cells (AECII)

The isolation, purification and culture of neonatal rat AECII cells were performed, as described previously (24–26). Briefly, the lungs of neonatal rats were removed within 24 h of birth, following anesthesia with 5% chloral hydrate (0.6 ml/100 mg; i.p.). The lungs were placed in cold D-Hank’s balanced buffer solution, and the trachea, main bronchus and hilar tissues were dissected and discarded; the remaining tissue was rinsed twice in D-Hank’s balanced buffer solution and cut into small pieces (~1 mm^3^). The lung tissue was incubated in 10 ml phosphate-buffered saline (PBS; Sigma-Aldrich) supplemented with 0.25% trypsin (Merck Millipore, Darmstadt, Germany) and 0.02% ethylenediaminetetraacetic acid, in a constant temperature water bath at 37°C with agitation for 30 min, in order to digest the tissue. The same volume of Dulbecco’s modified Eagle’s medium (DMEM; HyClone, Logan, UT, USA) containing 10% fetal bovine serum (FBS; HyClone) was added to terminate the digestion. The dissassociated cells were then filtered through a 200-mesh cell strainer, centrifuged at 71.5 × g for 5 min and the supernatant was discarded. Subsequently, 0.1% type I collagenase (Gibco Life Technologies, Carlsbad, CA, USA) in D-Hank’s balanced buffer solution was added to the re-suspended cells, which were digested for 20 min at 37°C. The digested cells were centrifuged, the supernatant was discarded and DMEM supplemented with 10% FBS was used to resuspend the cells. Fibroblasts were removed using the differential attachment procedure (repeated twice, 50 min/time). Unattached cells were transferred to 100 mm culture dishes coated with rat immunoglobulin G(IgG) (Abcam, Hong Kong, China), and the AECII cells were further purified, based on the IgG binding properties of the cells. Typan blue staining (Sigma-Aldrich) showed that >94% of purified AECII cells were viable. The purity of the isolated AECII cells was determined by calculating the positive perentage of immunofluorescence staining of surfactant protein (specific marker of AECII cells) in the isolated cells. The isolated AECII cells were cultured in DMEM containing 10% FBS, 100 U/ml penicillin and 100 mg/ml streptomycin (Life Technologies, Rockville, MD, USA) at 37°C in an atmosphere containing 21% O_2_ and 5% CO_2_. Following culture, the cell density was adjusted to 2–3×10^6^ cells/ml and a portion of the purified cells (0.4 ml) were seeded onto glass coverslips (15 mm × 15 mm; WHEATON, Millville, NJ, USA) for immunofluorescence staining whilst others (2 ml) were seeded into Petri dishes (Thermo Fisher Scientific, Waltham, MA, USA) for analysis by competitive enzyme-linked immunosorbent assay (ELISA), comet assay, western blotting or reverse transcription quantitative polymerase chain reaction (RT-qPCR). The cells from each isolation were cultured with 21% O_2_ and 5% CO_2_ in an incubator (Thermo Fisher Scientific) for 24 h. The cultures were replaced with fresh medium to remove unattached cells and the attached cells were randomly divided into either a 90% O_2_/5% CO_2_-exposed hyperoxia group or a 21% O_2_/5% CO_2_-exposed normoxia control group. The cells were then cultured for 12, 24, 48 or 72 h at 37°C in an incubator (Thermo Fisher Scientific) and were subsequently collected for the corresponding experiments. The purity of the AECII cells was ~90–95% following 1 day of culture. The AECII cells were identified using three methods: Inverted phase contrast microscopy, transmission electron microscopy (TEM) and surfactant protein-C (SP-C) detection by immunofluorescence staining.

### Detection of 8-hydroxy-2′-deoxyguanosine (8-OHdG)

A competitive ELISA for 8-OHdG was performed using a commercial 8-OHdG ELISA kit (Cayman Chemicals Co., Ann Arbour, MI, USA), according to the manufacturer’s instructions. The DNA was purified from the lung tissues and AECII cells using a Wizard Genomic DNA Purification kit (Promega Corporation, Madison, WI, USA) and the DNA purity was confirmed by measuring the A260:A280 ratio. Enzymatic digestion was performed using nuclease P1 (pH 5.3; Sigma-Aldrich) at 50°C for 1 h and with alkaline phosphatase (pH 8.5; Sigma-Aldrich) at 37°C for 30 min. The samples were boiled for 10 min and placed on ice for 5 min. The DNA hydrolysates were analyzed by ELISA, according to the manufacturer’s instructions. The plates were read at a wavelength of 412 nm (Tecan Sunrise Microplate reader; Tecan, Männedorf, Switzerland) and the level of 8-OHdG was determined for each sample from a standard curve.

### Comet assay

A Comet Assay kit (Cell Biolabs, Inc., Beijing, China) was used for single cell gel electrophoresis, according to the manufacturer’s instructions. Briefly, 1×10^6^ cells/ml AECII cells were washed with PBS and the cell suspension was mixed with liquified agarose at a 1:9 (v/v) ratio. A small aliquot of this mixture (100 *μ*l) was immediately transferred to agarose-coated slides (Thermo Fisher Scientific) and lysed (Cell Lysis Solution; Sigma-Aldrich) at 4°C in the dark for 2 h. Following cell lysis, the slides were treated with alkaline solution containing 0.6 mM Na-EDTA and 0.18 M NaOH (pH 13) at room temperature in the dark for 30 min to unwind the double-stranded DNA. The slides were electrophoresed (Biolab Comet-061; Beijing Biolab Science and Technology Co., Ltd, Beijing, China) under alkaline conditions at room temperature at 1 V/cm for 20 min. and subsequently neutralized using 0.4 M Tris (pH 7.5; Sigma-Aldrich) and fixed with absolute ethanol (100%). Following 10 min staining with SYBR Green dye (200 *μ*l; Biotium, Inc., Hayward, CA, USA), images were captured by fluorescence microscopy using a Nikon E2000 Microscope system and the accompanying software (Nikon Corporation, Tokyo, Japan). Images were scored for comet assay parameters, including tail length, olive tail moment and the percentage of DNA in the tail, using Comet A v.1.0 image analysis software (Cells Biolab, Inc., Beijing, China).

### Immunofluorescence staining

The left lobes of the lungs were inflated using 4% paraformaldehyde, soaked in 3% paraformaldehyde for 3 h at 4°C, cryoprotected in 30% sucrose (Sigma-Aldrich) for 12 h at 4°C and then frozen at −80°C. The frozen sections (8 *μ*m thick) were air-dried and washed three times with PBS. The AECII cells were fixed using 4% paraformaldehyde (Sigma-Aldrich) for 30 min and were washed three times with PBS. The sections were incubated with 0.3% Triton X-100 (Sigma-Aldrich) for 5 min at room temperature and washed three times with PBS. The sections were then blocked with 5% goat serum for 1 h at room temperature and incubated with goat polyclonal anti-OGG1 primary antibody (1:50; ab115841; Abcam) overnight at 4°C. Sections incubated in the absence of the primary antibodies were used as negative controls. The tissue sections were washed three times with PBS and incubated with tetramethylrhodamine isothiocyanate-conjugated mouse anti-goat immunoglobulin G secondary antibody (1:100; sc-3916; Santa Cruz Biotechnology, Inc., Santa Cruz, CA, USA) for 1 h at 37°C. The sections were washed three times with PBS and the nuclei were stained with 4′,6-diamidino-2-phenylindole dihydrochloride (1:2,000; Sigma-Aldrich) for 2 min. The slides were subsequently washed three times with PBS and images were captured using an MTC-600 confocal laser scanning microscope (Bio-Rad Laboratories, Inc., Hercules, CA, USA).

### Western blotting

The lung tissues or AECII cells were homogenized in radioimmunoprecipitation assay lysis buffer with phenylmethanesulfonyl fluoride (Sigma-Aldrich). Following centrifugation at 15,000 × g for 10 min at 4°C, the protein lysates in the supernatant were quantified using a Bicinchoninic Acid Protein Assay kit (Beyotime Institute of Biotechnology, Shanghai, China). The proteins were loaded and separated through a 10% SDS-polyacrylamide gel and were transferred onto polyvinylidene fluoride membranes (Merck Milipore, Boston, MA, USA). The membranes were blocked in 5% non-fat milk dissolved in Tris-buffered saline (TBS) for 2 h at room temperature prior to incubation overnight at 4°C with anti-OGG1 (1:400; Abcam) and anti-β-actin primary antibodies (1:1,000; Santa Cruz Biotechnology, Inc.). The membranes were then washed in TBS-0.2% Tween 20 (TBST) and incubated with horseradish peroxidase-conjugated secondary antibody at 37°C for 2 h. The membranes were washed in TBST, as previously. An enhanced chemiluminescence detection kit (EMD Millipore, Billerica, MA, USA) and a WD-9413B Gel Imaging system (Liuyi Instrument Factory, Beijing, China) were used for chemiluminescence analysis and imaging. The bands were quantified using ImageJ 1.45s software (National Institutes of Health, Bethesda, MD, USA) and the optical densities of all bands were normalized to those of β-actin.

### RT-qPCR

Total RNA was purified from lung tissues or the AECII cells using TRIzol reagent (Invitrogen Life Technologies, Carlsbad, CA, USA), according to the manufacturer’s instructions. The RNA samples were treated with 10 *μ*l DNase for 30 min (Takara Biotechnology Co., Dalian, China), according to the manufacturer’s instructions. The RNA purities were confirmed by measuring the A260:A280 ratio and an aliquot of total RNA (1 *μ*g) per sample was reversed-transcribed to cDNA using a PrimeScript RT reagent kit (Takara Biotechnology Co.), according to the manufacturer’s instructions. RT-qPCR was performed on an ABI PRISM 7900HT system (Applied Biosystems Life Technologies, Foster City, CA, USA) using equal volumes of cDNA (2*μ*l) with a SYBR Premix Ex Taq II kit (Takara Biotechnology Co.), according to the manufacturer’s instructions. PCR was performed using specific primer pairs (Table 1), according to the following program: 95°C for 30 sec; 40 cycles of 95°C for 5 sec and 60°C for 34 sec; 95°C for 15 sec; 60°C for 1 min and 95°C for 15 sec. Melting curve analyses were performed for the amplified genes and the specificity and integrity of the PCR products were confirmed by the presence of a single peak. For determination of the relative gene expression levels, the target mRNA were amplified using the same procedure and the expression levels were calculated relative to β-actin using the 2^−ΔΔCt^ method, as described previously (27). in the Applied Biosystems User Bulletin #2 ‘Relative quantification of gene expression’.

### Statistical analysis

Statistical analysis was performed using SPSS 17.0 software (SPSS, Inc., Chicago, IL, USA). Data were analyzed by one-way analysis of variance with a Bonferroni post-hoc test. P<0.05 was considered to indicate a statistically significant difference.

## Results

### Identification of cultured neonatal rat AECII cells

**Figure 1:**
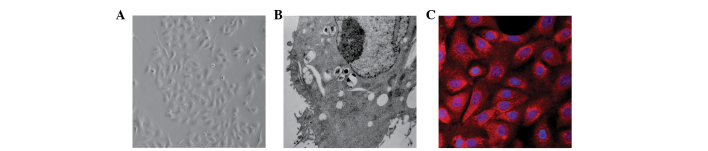
Identification of cultured neonatal rat alveolar epithelial type II cells. (A) Inverted phase contrast microscopy (magnification, ×200). (B) Transmission electron microscopy (magnification, ×5,000). (C) Surfactant protein-C detected by immunofluorescence staining (magnification, ×400).

The cultured neonatal rat AECII cells were identified using inverted phase contrast microscopy (Fig. 1A), TEM (Fig. 1B) and SP-C detection using immunofluorescence staining (Fig. 1C).

### Effects of hyperoxia exposure on the expression of 8-OHdG in neonatal rat lung tissues and cultured AECII cells

**Figure 2:**
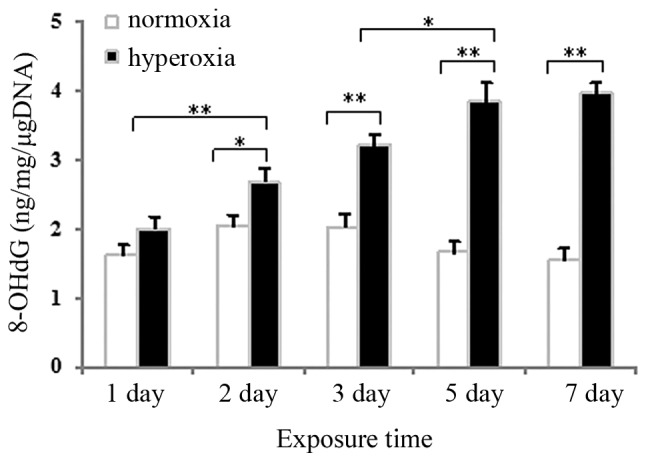
Hyperoxia-induced DNA damage in neonatal rat lung tissues. Competitive enzyme-linked immunosorbent assay was used to detect 8-OHdG in the rat lung tissues. Data are expressed as the mean ± standard deviation of the mean (n=6 per group; ^*^P<0.05; ^**^P<0.01 as compared with the normoxia group). 8-OHdG, 8-hydroxy-2′-deoxyguanosine.

**Figure 3:**
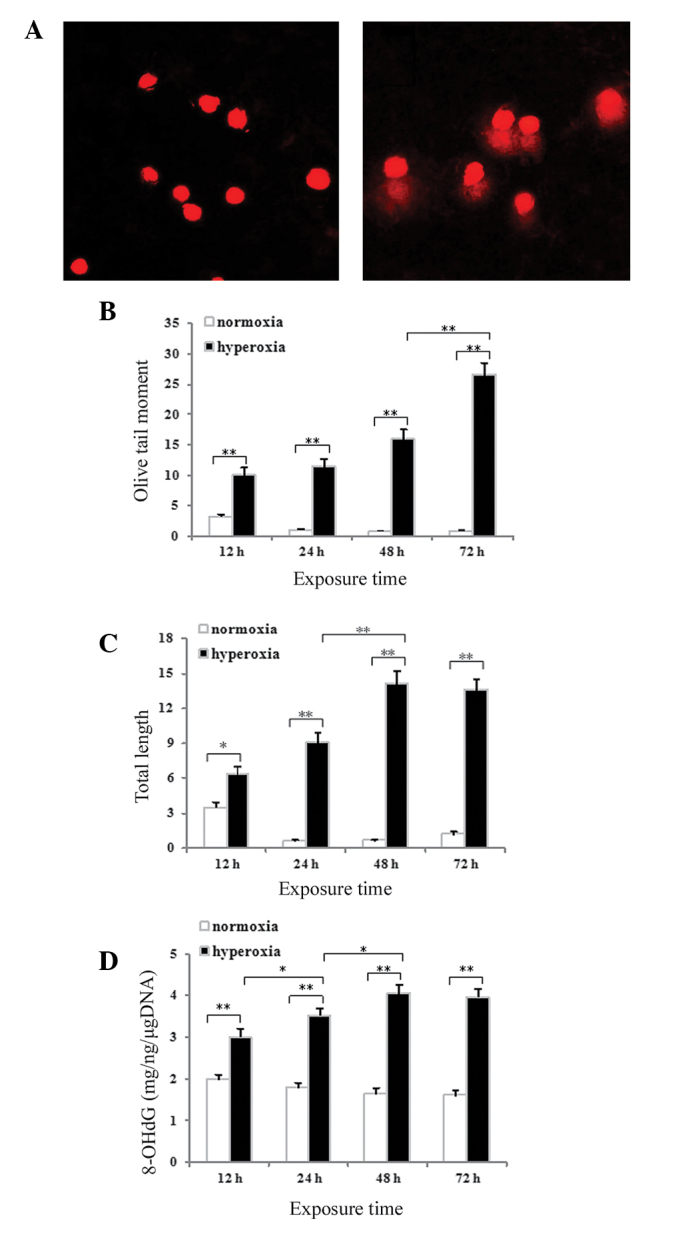
Hyperoxia-induced DNA damage in cultured AECII cells. (A) Fluorescence of ‘comets’ in alveolar epithelial type II cells under normoxia and hyperoxia for 48 h (magnification, ×400), following which the (B) olive tail moments, (C) tail lengths and (D) 8-OHdG DNA were quantified. Data are expressed as the mean ± standard deviation (^*^P<0.05 and ^**^P<0.01 as compared with the normoxia group). 8-OHdG, 8-hydroxy-2′-deoxyguanosine.

Competitive ELISA was used to measure 8-OHdG, a marker of oxidative DNA damage, in DNA samples from neonatal rat lung tissues and in cultured AECII cells exposed to hyperoxia or normoxia. As shown in Fig. 2, no significant change in the expression of 8-OHdG was observed after 1 day of hyperoxia exposure, compared with the normoxia group (P>0.05), whereas the expression of 8-OHdG in the lung tissues exposed to hyperoxia for 2, 3, 5 or 7 days significantly increased (P<0.05 for 2 days and P<0.01 for 3, 5 and 7 days). As shown in Fig. 3E, hyperoxia exposure was associated with significantly increased expression of 8-OHdG in the AECII cells at all time-points compared with the normoxia control (P<0.01) The 8-OHdG content in the lung tissues and cultured AECII cells increased gradually as the hyperoxia exposure time increased.

### Hyperoxia-induced DNA strand breaks in cultured neonatal rat AECII cells

An alkaline comet assay was used to assess DNA strand breaks in the AECII cells. Minimal migration of DNA was detected following exposure to normoxia for 48 h (Fig. 3A). By contrast, significant migration of the DNA from the nucleus, forming a comet tail, was observed following exposure to hyperoxia for 48 h (Fig. 3B). Measurements of comet tail length and olive tail moment were used to evaluate the DNA strand breaks. The tail length (P<0.05 for 12 h and P<0.01 for 24, 48 and 72 h; Fig. 3C) and the olive tail moment (P<0.01 for 12, 24, 48 and 72 h; Fig. 3D) increased significantly with time in the hyperoxia group compared with the normoxia control group.

### Effects of hyperoxia exposure on the localization and expression of OGG1 protein in neonatal rat lung tissues and cultured AECII cells

**Figure 4:**
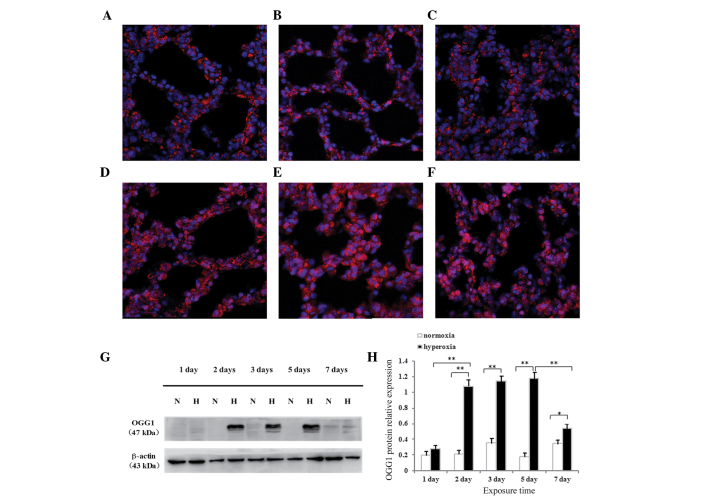
Expression of OGG1 (red stain) is predominantly localized in the cytoplasm on (A) day 1 and (B) day 5 in normoxia-exposed rats and on (C) day 1 in hyperoxia-exposed rats. On days (D) 3, (E) 5 and (F) 7 in hyperoxia (magnification, ×400), the expression of OGG1 increased in the cytoplasm and the nucleus. (G and H) Western blotting revealed similar patterns of expression. Data are expressed as the mean ± standard deviation (^*^P<0.05, ^**^P<0.01 as compared with the normoxia group). OGG1, 8-oxoguanine DNA glycosylase 1; N, normoxia; H, hyperoxia.

The expression and localization of OGG1 protein in the alveolar epithelium of newborn rats were determined using immunofluorescence confocal microscopy. Following normoxia exposure for 1 day (Fig. 4A) or 5 days (Fig. 4B), or hyperoxia exposure for 1 day (Fig. 4C), OGG1 was located primarily in the cytoplasm, and no difference was observed between the hyperoxia and normoxia groups. Following hyperoxia exposure for 3 days (Fig. 4D), 5 days (Fig. 4E) or 7 days (Fig. 4F), the localization of OGG1 significantly increased in the nucleus and, particularly in the cytoplasm. The protein expression of OGG1 was highest following hyperoxia exposure for 3 or 5 days. The protein expression of OGG1 was significantly reduced 7 days after hyperoxia, compared with 3 and 5 days, however, the expression remained elevated compared with that observed on day 1.

**Figure 5:**
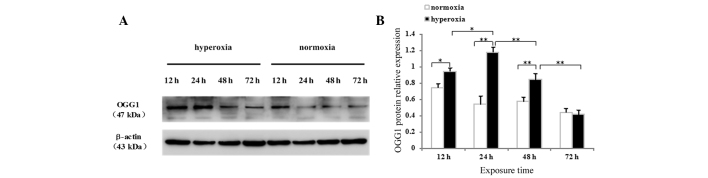
Protein expression of OGG1 in cultured neonatal rat alveolar epithelial type II cells. (A) Western blotting and (B) densitometric quantification of the protein expression of OGG1 following different durations of hyperoxia or normoxia exposure. Data are expressed as the mean ± standard deviation (^*^P<0.05; ^**^P<0.01 as compared with the normoxia group). OGG1, 8-oxoguanine DNA glycosylase 1.

The present study also detected the protein expression of OGG1 in neonatal rat lung tissues and cultured AECII cells by western blotting. The OGG1 protein was identified as a dominant band of ~47 kDa in the lung tissues (Fig. 4G) and AECII cells (Fig. 5A) exposed to hyperoxia or normoxia at all time-points. As shown in Fig. 4F, no difference in the protein expression of OGG1 was observed after 1 day of hyperoxia exposure compared with the controls (P>0.05), whereas the protein expression of OGG1 in the lung tissues exposed to hyperoxia for 2, 3, 5 or 7 days was significantly increased (P<0.01 for 2, 3 and 5 days; P<0.05 for 7 days). The protein expression of OGG1 in the lung tissues began to increase following 2 days of hyperoxia exposure, peaked between 3 and 5 days and began to decrease following 7 days of hyperoxia. As shown in Fig. 5B, the protein expression of OGG1 increased significantly in cells exposed to hyperoxia for 12, 24 and 48 h, compared with the controls (P<0.05 for 12 h; P<0.01 for 24 and 48 h), whereas no difference was observed in the protein expression of OGG1 72 h after hyperoxia exposure (P>0.05). The protein expression of OGG1 in the hyperoxia-exposed AECII cells began to increase after 12 h, peaked after 24 h, began to decrease after 48 h and decreased further after 72 h.

### Effects of hyperoxia exposure on the mRNA expression of OGG1 in neonatal rat lung tissues and neonatal rat AECII cells

**Figure 6:**
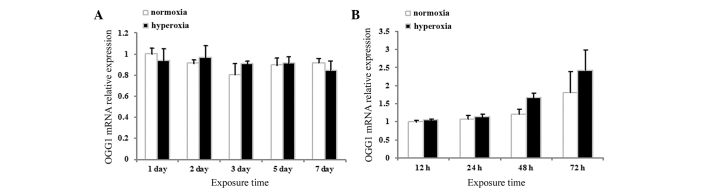
mRNA expression levels of OGG1 in lung tissues and AECII cells. (A) Neonatal rat lung tissues and (B) neonatal rat AECII cells exposed to hyperoxia or normoxia. Data are expressed as the mean ± standard deviation (P>0.05 for all comparisons). AECII, alveolar epithelial type II cells; OGG1, 8-oxoguanine DNA glycosylase 1.

The mRNA expression levels of OGG1 in the neonatal rat lung tissues and cultured AECII cells were assessed using RT-qPCR. No significant differences were observed between the mRNA expression levels of OGG1 in the hyperoxia and normoxia group in either the lung tissues (P>0.05; Fig. 6A) or the cultured AECII cells (P>0.05; Fig. 6B) at any of the time-points.

## Discussion

BPD is a multifactorial disease, however, oxidative stress resulting from multiple causes is the predominant pathogenic factor in BPD (28). Several previous studies have confirmed that oxidative stress-induced lung injury is involved in the occurrence and development of BPD (29,30). The expression of macrophages and interleukins are increased in the bronchial alveolar lavage fluid of children with BPD (31,32) and animal models of BPD exhibited increased levels of pro-inflammatory cytokines, including interleukin-1 and tumor necrosis factor-α (33). Inhalation of high oxygen concentrations can increase the lipid peroxidation of lung tissues and cause oxidative stress damage in the lungs (10–13,32). The importance of oxidative stress in BPD is well-established, and the prevention and treatment of pulmonary oxidative damage have been a focus of antioxidant therapies (34–36). However, the effects of treating pediatric patients with BPD with general antioxidants are insubstantial (34,37), therefore, a novel therapeutic approach for the prevention and treatment of BPD is imperative.

The most serious effect of oxidative stress is DNA damage. In pre-term baboons, mechanical ventilation with high concentrations of oxygen (100% O_2_) increased the level of oxidative DNA damage to lung tissues compared with controls receiving levels of oxygen required to maintain PO_2_ between 50 and 80 mmHg (38). Marked oxidative DNA damage occurs following exposure of A549 cells to 95% O_2_ and the damage increases gradually with increasing exposure duration (39). The present study confirmed that the occurrence of oxidative DNA damage was induced in the lung epithelium of newborn rats by exposure to continuously high concentrations of oxygen (90% O_2_). In addition, DNA damage was exacerbated with increased hyperoxia exposure duration. These findings are consistent with previous studies, demonstrating that hyperoxia increases DNA damage (23,38,39).

ROS attack nuclear and mitochondrial DNA, inducing various DNA mutations (40). BER is regarded as the predominant DNA repair pathway, in which DNA glycosylase-mediated identification and the excision of damaged bases occurs (15,16). OGG1 is the most important BER enzyme involved in this step (18,19). In nuclear cataracts developed in adult Wistar rats exposed to 60% oxygen, DNA damage in the lens increases concurrent with an increased protein expression of OGG1 (41). Exposure of A549 cells to high oxygen levels significantly increases the level of DNA damage and overexpression of OGG1 in A549 cells, and adult rat AECII cells can alleviate DNA damage and increase the survival rate of cells exposed to hyperoxia (39). OGG1 is an established antagonist of DNA damage caused by oxidative stress, however, the association between OGG1 and the development of hyperoxia-induced BPD remains to be elucidated.

The present study used *in vitro* and *in vivo* hyperoxia exposure experiments and confirmed that the protein expression of OGG1 increased as the duration of hyperoxia exposure increased during early-stage hyperoxia exposure, with an increased in OGG1 in the cytoplasm. Following a peak in expression, the protein expression of OGG1 decreased with increasing duration of hyperoxia exposure. High oxygen potentially induced severe oxidative DNA damage in the pulmonary epithelium, thereby activating the DNA repair protein, OGG1. With extended periods of hyperoxia exposure, the reduction in the protein expression of OGG1 may account for a concomitant suppression of DNA damage repair, causing DNA damage to accumulate. The upregulation of OGG1 was associated with an increased level of cytoplasmic OGG1. Therefore, it was hypothesized that localization of OGG1 to the cytoplasm is important in the occurrence of BPD. With its proximity to the electron transport chain and the relatively limited mitochondrial DNA repair capacity, mitochondrial DNA is significantly more sensitive to ROS-mediated oxidative DNA damage, compared with nuclear DNA (42,43). Previous studies have suggested that mitochondrially localized OGG1 is involved in antagonizing the oxidative DNA damage induced by ROS (20,21,44). The *in vitro* and *in vivo* analyses performed in the present study indicated no significant increase in the mRNA expression of OGG1 at any time-point in the hyperoxia-exposed groups. The mRNA expression of OGG1 was not consistent with the protein expression of OGG1, therefore, the present study hypothesized that OGG1 is regulated primarily at the level of protein expression during the occurrence of hyperoxia-induced BPD. The induction of oxidative DNA damage in the spleens of adult Sprague Dawley rats exposed to subchronic aniline was associated with significant increases in the protein and mRNA expression levels of OGG1 (45), however, this is not entirely consistent with the results of the present study.

The present study demonstrated that severe DNA damage occurred in lung epithelial cells during early-stage BPD. The DNA repair gene, OGG1, may be important in this process and further investigations are being performed to elucidate the underlying regulatory mechanisms of OGG1 during hyperoxia-induced BPD.

## Figures and Tables

**Table I:** Primers used for reverse transcription quantitative polymerase chain reaction.

Gene	Forward (5′–3′)	Reverse (5′–3′)
OGG1	CTAAGAAGACAGAAGGCTAGGTAG	TGACTTTGATTTGGGATGTTTGC
β-actin	CGTGCGTGACATTAAAGAG	TTGCCGATAGTGATGACCT

OGG1, 8-oxoguanine DNA glycosylase 1.
